# Yangyinghuoxue decoction exerts a treatment effect on hepatic fibrosis by PI3K/AKT pathway in rat model: based on the network pharmacology and molecular docking

**DOI:** 10.18632/aging.205559

**Published:** 2024-02-15

**Authors:** Yan-Ming Bai, Shuang Liang, Bo Zhou

**Affiliations:** 1 School of Traditional Chinese Medicine, Ningxia Medical University, Yinchuan 750004, China; 2 Yinchuan Hospital of Traditional Chinese Medicine, Ningxia Medical University, Yinchuan 750001, China; 3 Ningxia Regional Key Laboratory of Integrated Traditional Chinese and Western Medicine for Prevention and Treatment of High Incidence, Ningxia Medical University, Yinchuan 750004, China

**Keywords:** Yangyinghuoxue decoction (YYHXD), hepatic fibrosis (HF), network pharmacology, PI3K-Akt signaling pathway, molecular docking

## Abstract

Background: Yangyinghuoxue decoction (YYHXD) is a Traditional Chinese medicine (TCM) compound with satisfactory clinical efficacy in the treatment of hepatic fibrosis (HF). However, the pharmacological molecular mechanisms of YYHXD in the treatment of hepatic fibrosis have not yet been clarified.

Objective: To determine the pharmacological mechanisms of YYHXD for the treatment of hepatic fibrosis via network pharmacology analysis combined with experimental verification.

Methods: First, the bioactive ingredients and potential targets of YYHXD and HF-related targets were retrieved from the online databases and literatures. Next, the “herb-ingredient-target-disease” network and PPI network were constructed for topological analyses and key active compounds and targets screening. Enrichment analyses were performed to identify the critical biological processes and signaling pathways. Then, the molecular docking experiment was performed to initially validate the network pharmacology prediction results. Finally, the antifibrotic effect and pharmacological mechanisms of YYHXD were investigated in CCl_4_ induced liver fibrosis in rats.

Results: In total, 141 active compounds in YYHXD, 637 YYHXD-related targets and 1598 liver fibrosis-related targets were identified. Among them, 69 overlapped targets were finally obtained. Network analysis screened 5 critical bioactive components and 34 key targets. Functional enrichment analysis indicated that YYHXD obviously influenced biological processes such as oxidative stress, cellular inflammation and hepatocyte apoptosis and signaling pathways such as PI3K-Akt, Apoptosis, and JAK-STAT in the treatment of HF. The molecular docking results suggested that the YYHXD may have a direct impact on the PI3K-Akt signaling pathway. Further, *in vivo* experiment indicated that YYHXD treatment not only reduced liver injury and protected liver function, but also decrease the apoptosis of hepatic parenchyma cells, reducing inflammatory and attenuating oxidative stress. Moreover, YYHXD significantly attenuated the upregulation of target proteins enriched in PI3K signaling pathway, including P-PI3K, P-Akt1, HSP90, MYC, p53.

Conclusions: The mechanisms of YYHXD against liver fibrosis were involved in multiple ingredients, multiple targets and multiple signaling pathways. The PI3K/Akt pathway could be the most important pharmacological mechanism of YYHXD therapy for liver fibrosis.

## INTRODUCTION

Hepatic Fibrosis (HF) is a repair response of the body to chronic liver damage caused by hepatitis virus, alcohol, metabolic and genetic, immune abnormalities and other etiologies [1]. It is mainly manifested as excessive proliferation and deposition of extracellular matrix (ECM) in liver tissue, which leads to abnormal changes in liver tissue structure and disrupts liver physiological functions [1, 2]. It is a serious public health problem that results in liver cirrhosis, liver cancer, and death [2, 3]. In recent years, the incidence of liver fibrosis has remained high in China and worldwide, expanding to all stages of the population and showing an increasing trend year by year [3–5]. Currently, no antifibrotic medications have been clinically approved for human use [6]. Hence, developing efficacious and safe anti-hepatic fibrosis agents and elucidating their mechanisms of action are of tremendous social value.

Traditional Chinese Medicine (TCM) has its unique advantages in the treatment of liver fibrosis. Its advantages of dialectical treatment, mild action, less adverse effects, lower medical costs and significant efficacy are becoming more and more prominent, and has become a hot spot in recent years for the diagnosis and treatment of liver fibrosis at home and abroad [7]. Studies have shown that TCM can exert therapeutic effects on liver fibrosis by inhibiting hepatic stellate cell activation, liver inflammatory response, extracellular matrix synthesis, promoting hepatic stellate cell apoptosis and protecting hepatocytes [7, 8]. Therefore, it is of the utmost importance to search for effective anti-liver fibrosis drugs from the treasury of TCM.

The TCM compound Yangyinghuoxue decoction (YYHXD), created based on the febrile disease theory, has long-term clinical and real-world experience in the treatment of liver fibrosis. YYHXD consists of fourteen herbs: *Radix Paeoniae Alba, Radix Bupleuri, tangerine peel, radix paeoniae rubra, Ligusticum chuanxiong, rhubarb, jujube, Salvia miltiorrhiza, angelica, Scutellaria baicalensis, licorice, Rehmannia glutinosa, Eupatorium adenophorum, Fritillaria thunbergii*. Our previous study showed that YYHXD could improve liver fibrosis in rats [9], but the pharmacological mechanisms of YYHXD therapy for liver fibrosis are not yet known.

Network pharmacology is a new discipline integrating systems biology, multidirectional pharmacology, computer analysis, network analysis and other disciplines. Network pharmacology, with the characteristics of integrity, systematicness and novelty, provides a new method to reveal the complex pharmacological mechanism of TCM [10, 11].

In this study, we applied network pharmacology approaches to investigated the potential pharmacological mechanisms of YYHXD treating liver fibrosis. We then validated the network pharmacology predictions through molecular docking simulations and animal experiments. This study represents the first effort to elucidate the potential mechanisms underlying YYHXD’s therapeutic effects on liver fibrosis by integrating network pharmacology analysis with experimental verification.

## MATERIALS AND METHODS

### Collection of the potential action targets of YYHXD and HF

The active compounds of each herb in YYHXD were retrieved from the TCMSP database (https://old.tcmsp-e.com/tcmsp.php) and published literature. Compounds from the TCMSP database were filtered for oral bioavailability (OB) ≥30% and drug-likeness (DL) ≥0.18. The potential targets of the YYHXD active compounds were identified using SwissTargetPrediction database (http://swisstargetprediction.ch/) and DrugBank database (https://www.drugbank.ca/).

The HF-related targets were obtained from the reported literatures and Online Mendelian Inheritance in Man (OMIM) database (http://www.omim.org/) and Human Phenotype Ontology (HPO) database. The overlapping genes between YYHXD-related targets and HF-related targets were considered potential YYHXD targets for treating HF and subjected to further analysis. A “herb-ingredient-target-disease” network of YYHXD was then established using Cytoscape software (version 3.7.2) based on the drug-disease overlapping targets and their corresponding active ingredients and corresponding herbs. The topological parameters of each node in the “herb-ingredient-target-disease” network were calculated using Cytoscape software. The five compounds with the highest degree score were screened out, which may be the key effective ingredients of YYHXD for treating liver fibrosis.

### Protein - protein interaction (PPI) network construction

The protein-protein interaction (PPI) analysis of overlapping targets was performed using the BisoGenet plugin in Cytoscape software. After screening twice with degree ≥2-fold median, the PPI network was constructed. The CytoHubba plugin was utilized to identify the hub genes from the PPI network based on the Maximal Clique Centrality (MCC) algorithm. The top 10 genes with the highest MCC scores were screened out as hub genes, which were considered as key targets for the treatment of hepatic fibrosis with YYHXD. Subsequently, the Molecular Complex Detection (MCODE) plugin in Cytoscape software was used to analyze PPI network modules. The PPI network was divided into several clusters based on the MCODE score. The cluster with the highest MCODE score was considered as the most critical module in the PPI network.

### Functional enrichment analysis

The DAVID database (https://david.ncifcrf.gov/tools.jsp) provides dependable algorithms for functional annotation, gene categorization, and identifier translation. To further decipher the potential biological processes and signaling pathways related to YYHXD’s therapeutic effects against liver fibrosis, we conducted Gene Ontology (GO) and Kyoto Encyclopedia of Genes and Genomes (KEGG) enrichment analyses utilizing DAVID tools on modularized genes from the highest-ranked protein association module. The GO analysis delineates genes into three domains: Biological Processes (BP), Cellular Components (CC), and Molecular Functions (MF).

### Molecular docking verification of the key ingredient and target

The five key active compounds from the “herb-ingredient-target-disease” network were docked against proteins involved in the PI3K pathway. The 3D structures of proteins and ligands were obtained from the PDB (https://www.rcsb.org) and PubChem (https://pubchem.ncbi.nlm.nih.gov/) databases, respectively. Molecular docking simulations were performed in Discovery Studio 2019 to validate the preceding enrichment analysis predictions. Briefly, protein and ligand structures were loaded into Discovery Studio and subjected to LibDock docking with parameters of 100 hotspots and 0.25Å tolerance. The docking program was executed and results visualized using Discovery Studio tools. Binding affinities between active compounds and target proteins were evaluated by LibDock scores.

### Reagents and antibodies

CCl4 was purchased from Bodi Chemical Co., Ltd., (Tianjin, China). Olive oil was purchased from Beijing Solarbio Science and Technology Co., Ltd., (Beijing, China). Colchicine and pentobarbital sodium were obtained from Wuhan Servicebio Technology Co., Ltd., (Wuhan, China). Assay kits for aspartate aminotransferase (AST), alanine aminotransferase (ALT), interleukin 6 (IL6), tumor necrosis factor α (TNF-α) kits, hyaluronic acid (HA) and laminin (LN) were purchased from Shanghai Enzyme Research Biotechnology Co., Ltd., (Shanghai, China). Kits for superoxide dismutase (SOD), glutathione peroxidase (GSH-Px) and malondialdehyde (MDA) were purchased from Nanjing Jiancheng Biological Engineering Research Institute (Nanjing, China). The TUNEL Apoptosis Assay Kit was purchased from Thermo Fisher Scientific (Waltham, MA, USA). BCA Protein Assay Kit was purchased from Abcam (Cambridge, MA, USA). The chemiluminescent substrate (ECL kit) was purchased from Affinity (Jiangshu, China). Antibodies against P-PI3K, P-Akt, HSP90, MYC, p53, α-SMA, collagen I and β-actin were from Affinity (Jiangshu, China). Secondary antibodies were from Affinity (Jiangshu, China).

### Preparation of prescriptions’ extract

The YYHXD is composed of 14 crude drug materials, including *Radix Paeoniae Alba, Radix Bupleuri, tangerine peel, radix paeoniae rubra, Ligusticum chuanxiong, rhubarb, jujube, Salvia miltiorrhiza, angelica, Scutellaria baicalensis, licorice, Rehmannia glutinosa, Eupatorium adenophorum* and *Fritillaria thunbergii* as a fixed ratio of 4:5:5:5:5:3:3:4:4:4:3:4:3:3. All the herbs were provided by School of Traditional Chinese Medicine, Ningxia Medical University.

The herbal prescription was decocted twice by heating under reflux with 8 volumes of water for 1 hour each time. The decoction was then filtered, the filtrates combined, and concentrated to 1.0 g crude drug per mL for subsequent administration.

### Animals and drug administration

A total of 48 Wistar male rats (200 ± 10 g) were obtained from the Laboratory Animal Center of Ningxia Medical University. After obtaining IACUC approval (IACUC-NYLAC-2021-009), the rats were housed with ad libitum access to food and water under standard conditions (22–24°C, 45–50% humidity, 12-h dark/illumination cycle). Following 7 days of acclimatization, the rats were randomly allocated into four groups (*n* = 12 per group): normal control, CCl4 model, YYHXD treatment, and colchicine positive control. Except for the normal control group, hepatic fibrosis was triggered in the rats by administering CCl_4_ subcutaneously (diluted in 40% olive oil) twice per week for 6 weeks. The normal control group received olive oil only. In week 7, the YYHXD and colchicine groups were gavaged with YYHXD (10 mL/kg) and colchicine suspension (0.2 mg/10 mL/kg) respectively for 6 weeks. The control and CCl4 groups were sham-gavaged with distilled water (10 mL/kg). At 24 h after the final gavage, all rats were anesthetized with 3% sodium pentobarbital to collect blood samples from the inferior vena cava and liver tissues. This animal study was conducted in accordance with the research ethics guidelines and animal management regulations of the Chinese Ministry of Health.

### Histological analysis

The liver tissues were fixed in 10% neutral buffered formalin for 24 h, followed by paraffin embedding and sectioning at 5 μm thickness. The tissue sections were subjected to hematoxylin and eosin (H&E) staining for histopathological examination and Masson’s trichrome staining for collagen deposition analysis to evaluate liver injury and fibrosis.

### Serum biochemical analysis

Serum biomarkers aspartate aminotransferase and alanine aminotransferase were quantified by colorimetric assays to assess hepatocyte damage. As cytoplasmic enzymes, their elevated levels indicate liver injury. Pro-inflammatory cytokines interleukin-6 and tumor necrosis factor-α in serum were measured by ELISA kits to evaluate inflammation status. Serum hyaluronic acid and laminin were determined by ELISA as indicators of liver fibrosis progression. They are extracellular matrix components that accumulate with increasing fibrotic tissues.

### Determination of liver oxidative stress biomarkers

The activities of antioxidant enzymes superoxide dismutase (SOD) and glutathione peroxidase (GSH-Px), along with the level of lipid peroxidation marker malondialdehyde (MDA) in liver were assayed colorimetrically using commercial kits following manufacturers’ guidelines.

### TUNEL assay

Terminal deoxynucleotidyl transferase dUTP nick end labeling (TUNEL) staining was performed using the Colorimetric TUNEL Apoptosis Assay Kit per manufacturer’s protocol. In brief, paraffin-embedded liver sections were deparaffinized, digested with protease K (37°C, 20 min), rinsed, and incubated with 50 μL TUNEL reaction mixture (37°C, 60 min, dark). After DAPI counterstaining, fluorescence microscopy was used to evaluate apoptosis.

### Immunohistochemical staining

The paraffin-embedded liver tissues were cut into 4–6 mm slices, dewaxed and serially dehydrated in ethanol. The sections were boiled while immersed in citrate buffer for antigen retrieval, then incubated in 3% hydrogen peroxide for 10 minutes to inactivate endogenous peroxidase. The sections were incubated primary antibodies at 4°C overnight. The sections were then incubated with again secondary antibodies at room temperature for 20 min., stained with diaminobenzidine (DAB) and restained with hematoxylin. Positive signal was detected as a brown colour under a light microscope. Immunohistochemistry was used for assess the protein expression enriched in PI3K signaling pathway, including P-PI3K, P-Akt, HSP90, MYC, p53.

### Western blotting

Total protein was extracted from liver tissues using RIPA lysis buffer with protease inhibitors and quantified by BCA assay. Equal amounts of protein were separated by 11% SDS-PAGE, transferred to PVDF membranes, and blocked with 5% nonfat milk. The membranes were incubated overnight at 4°C with primary antibodies, followed by 1 h incubation with secondary antibodies at room temperature. Protein bands were visualized using ECL substrate and a gel imaging system. β-actin served as the loading control. Western blotting was performed to analyze the effects of YYHXD treatment on the protein levels of collagen I, α-SMA, and PI3K pathway components including p-PI3K, p-Akt, HSP90, MYC, and p53.

### Statistical analysis

Statistical analyses were performed using GraphPad Prism 8.3.0. Quantitative data are expressed as mean ± standard deviation (SD). Comparisons between two experimental groups were analyzed by Student’s *t*-test. One-way analysis of variance (ANOVA) followed by Dunnett’s multiple comparisons test was used to analyze the differences between multiple experimental groups. *P* < 0.05 was considered statistically significant.

### Data availability

All data, models, and code generated or used during the study appear in the submitted article.

## RESULTS

### Targets collection

**Figure 1:**
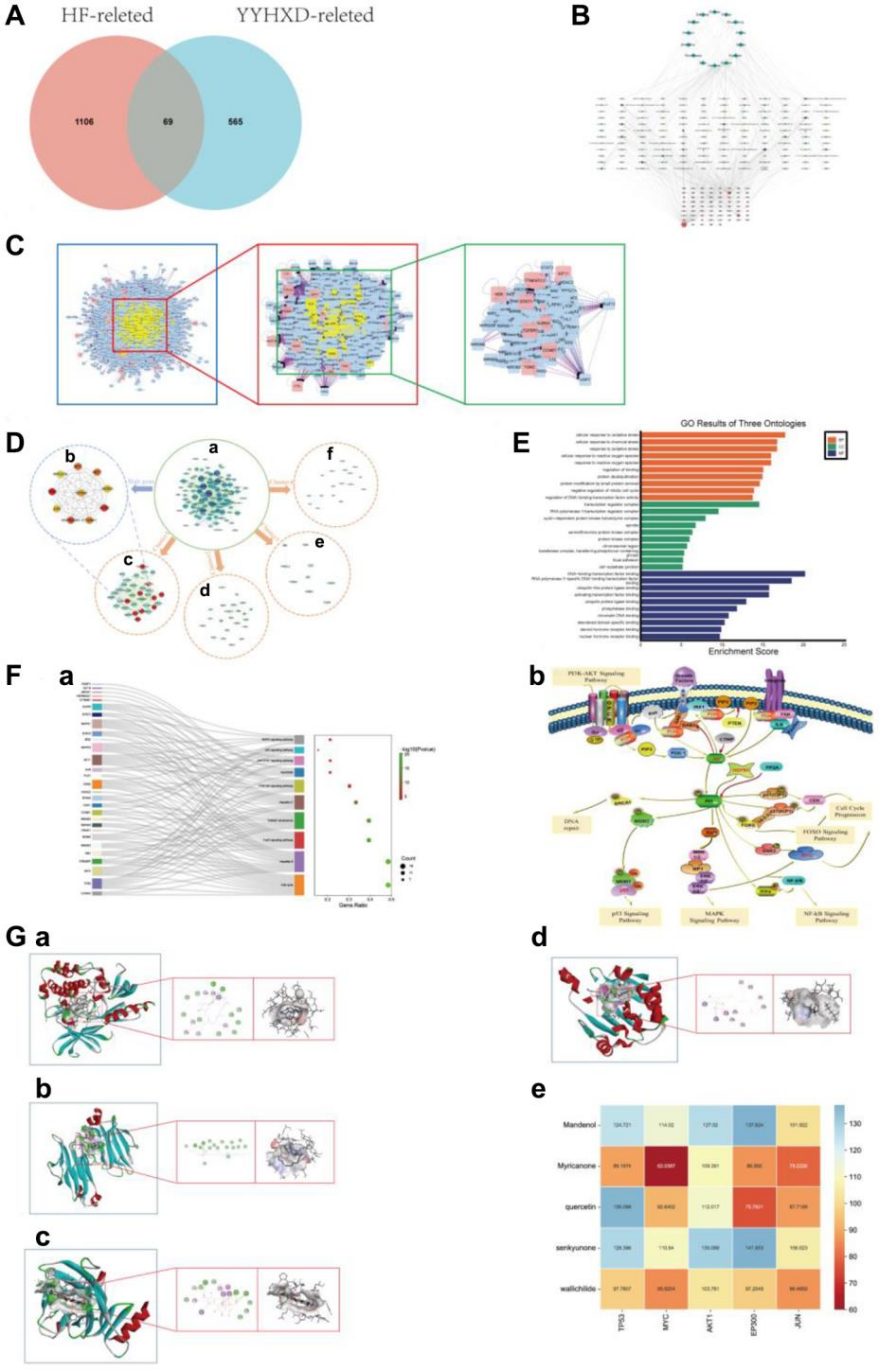
**Network pharmacology analysis.** (**A**) Intersection Venn diagram of YYHXD-HF. (**B**) The “herb - ingredient - target - disease” network. The purple nodes represented herb. The red nodes represented YYHXD-regulated targets, the node with larger size, represented higher degree score in the network. The green nodes represented active ingredients, the node with darker color, represented higher degree score in the network. (**C**) PPI network construction. (**D**) Gene cluster and core target analysis of PPI network. (**a**) PPI network. (**b**) Identification of hub genes in the PPI network. Nodes were ranked by MCC score, with darker red represents higher the MCC score. (**c**–**f**) 4 cluster genes identified in the PPI network. The clusters were ranked by MCODE score. Cluster 1 was the most critical module in the PPI network, with an MCODE score of 29.939, containing 34 nodes. The red nodes in cluster 1 represented hub genes of PPI network. Cluster 2 contained 22 nodes, with an MCODE score of 7.524. Cluster 3 contained 17 nodes, with an MCODE score of 3.875. Cluster 4 with an MCODE score of 3.5, contained 9 nodes. (**E**) GO annotation analysis of Cluster 1 (top 10 most significantly enriched GO terms in BP, CC and MF). (**F**) KEGG pathways enrichment analysis of Cluster 1. (**a**) The enrichment analysis of Cluster 1 (top 10 most significantly enriched KEGG pathways). (**b**) KEGG pathways. (**G**) Molecular docking results and visual analysis. (**a**) Docking visualization of AKT1 and senkyunone. (**b**) Docking visualization of MYC and mandenol. (**c**) Docking visualization of TP53 and quercetin1. (**d**) Docking visualization of HSP90AA1 and senkyunon. (**e**) The heat map of LibDock Scores between the 5 crucial active molecules and 4 hub targets.

Trough TCMSP database and literatures search, a total of 141 active ingredients of YYHXD were screened out (Table 1). Then, 634 corresponding targets were obtained from SwissTargetPrediction database and Drugbank database. Besides, 1175 related target genes for HF were collected from the online databases and literatures. The Venn diagram showed intersection of drug-related targets and HF-related targets (Figure 1A). From Venn diagram, 69 overlapping genes were identified.

**Table 1:** The active ingredients of YYHXD screened from TCMSP database.

**No**	**MOL**	**Compound**	**MW**	**OB**	**DL**	**Herb**
1	MOL001924	Paeoniflorin	480.51	53.87	0.79	BS
2	MOL000492	Catechin	290.29	54.83	0.24	BS, CS, DH, DZ, DS, HQ
3	MOL001645	Linoleyl acetate	308.56	42.10	0.20	CH
5	MOL004609	Areapillin	360.34	48.96	0.41	CH
6	MOL013187	Cubebin	356.40	57.13	0.64	CH
7	MOL004624	Longikaurin A	348.48	47.72	0.53	CH
8	MOL004653	(+)-Anomalin	426.50	46.06	0.66	CH
10	MOL000490	Petunidin	317.29	30.05	0.31	CH
4	MOL002776	Baicalin	446.39	40.12	0.75	CH, CS
9	MOL004718	α-spinasterol	412.77	42.98	0.76	CH, CS
11	MOL000098	Quercetin	302.25	46.43	0.28	CH, DZ
12	MOL005815	Citromitin	404.45	86.90	0.51	CP
13	MOL005100	5,7-dihydroxy-2-(3-hydroxy-4-methoxyphenyl)chroman-4-one	302.30	47.74	0.27	CP
14	MOL005828	nobiletin	402.43	61.67	0.52	CP
15	MOL004328	Naringin	272.27	59.29	0.21	CP
16	MOL007930	Hesperidin	610.62	13.33	0.67	CP
17	MOL006992	(2R,3R)-4-methoxyl-distylin	318.30	59.98	0.30	CS
18	MOL001494	Mandenol	308.56	42.00	0.19	CX
19		augustic-acid				CX
20	MOL002135	Myricanone	356.45	40.60	0.51	CX
21	MOL002151	senkyunone	326.52	47.66	0.24	CX
22	MOL002157	wallichilide	412.57	42.31	0.71	CX
23	MOL000357	Sitogluside	576.95	20.63	0.62	CX
24	MOL000433	FA	441.45	68.96	0.71	CX
25	MOL002173	3-N-butyl-4,5-dihydrophthalide	194.30	25.76	0.07	CX
26	MOL011782	Ligustilide	190.26	23.50	0.07	CX, DG
27	MOL002235	EUPATIN	360.34	50.80	0.41	DH
28	MOL002268	rhein	284.23	47.07	0.28	DH
29	MOL002281	Toralactone	272.27	46.46	0.24	DH
30	MOL000471	aloe-emodin	270.25	83.38	0.24	DH
74	MOL000414	Caffeic acid	180.17	54.97	0.05	DG
75	MOL000360	Ferulic Acid	194.20	39.56	0.06	DG
41	MOL001601	1,2,5,6-tetrahydrotanshinone	280.34	38.75	0.36	DS
42	MOL002222	sugiol	300.48	36.11	0.28	DS
43	MOL002651	Dehydrotanshinone II A	292.35	43.76	0.40	DS
44	MOL000006	luteolin	286.25	36.16	0.25	DS
45	MOL007045	3α-hydroxytanshinone II a	310.37	44.93	0.44	DS
46	MOL007049	4-methylenemiltirone	266.36	34.35	0.23	DS
47	MOL007059	3-beta-Hydroxymethyllenetanshiquinone	294.32	32.16	0.41	DS
48	MOL000338	3′-methyleriodictyol	302.30	51.61	0.27	DS
49	MOL000378	7-O-methylisomucronulatol	316.38	74.69	0.30	DS
50	MOL005406	atropine	289.41	45.97	0.19	DS
51	MOL001323	Sitosterol alpha1	426.80	43.28	0.78	DS
52	MOL007071	przewaquinone f	312.34	40.31	0.46	DS
53	MOL007152	przewaquinone E	312.34	42.85	0.45	DS
54	MOL000387	Bifendate	418.38	31.10	0.67	DS
55	MOL007107	C09092	286.50	36.07	0.25	DS
56	MOL007088	cryptotanshinone	296.39	52.34	0.40	DS
57	MOL007082	Danshenol A	336.41	56.97	0.52	DS
58	MOL007081	Danshenol B	354.48	57.95	0.56	DS
59	MOL007094	danshenspiroketallactone	282.36	50.43	0.31	DS
60	MOL007093	dan-shexinkum d	336.41	38.88	0.55	DS
61	MOL007098	deoxyneocryptotanshinone	298.41	49.40	0.29	DS
62	MOL007100	dihydrotanshinlactone	266.31	38.68	0.32	DS
63	MOL007101	dihydrotanshinone I	278.32	45.04	0.36	DS
64	MOL007058	formyltanshinone	290.28	73.44	0.42	DS
65	MOL008400	glycitein	284.28	50.48	0.24	DS
66	MOL000296	hederagenin	414.79	36.91	0.75	DS
67	MOL007108	isocryptotanshi-none	296.39	54.98	0.39	DS
68	MOL007111	Isofucosterol	294.37	49.92	0.40	DS
69	MOL000239	Jaranol	314.31	50.83	0.29	DS
70	MOL007111	Isotanshinone II	294.37	49.92	0.40	DS
71	MOL004576	taxifolin	304.27	57.84	0.27	DS
72	MOL007154	tanshinone iia	294.37	49.89	0.40	DS
73	MOL007156	tanshinone VI	296.34	45.64	0.30	DS
31	MOL012921	stepharine	297.38	31.55	0.33	DZ
32	MOL012976	coumestrol	268.23	32.49	0.34	DZ
33	MOL012992	Mauritine D	342.46	89.13	0.45	DZ
34	MOL001454	berberine	336.39	36.86	0.78	DZ
35	MOL001522	(S)-Coclaurine	285.37	42.35	0.24	DZ
36	MOL004350	Ruvoside_qt	390.57	36.12	0.76	DZ
37	MOL000627	Stepholidine	327.41	33.11	0.54	DZ
38	MOL007213	Nuciferin	295.41	34.43	0.40	DZ
39	MOL000787	Fumarine	353.40	59.26	0.83	DZ
40	MOL002773	beta-carotene	536.96	37.18	0.58	DZ
100	MOL001792	DFV	256.27	32.76	0.18	GC
101	MOL001484	Inermine	284.28	75.18	0.54	GC
102	MOL002311	Glyceyrol	366.39	90.78	0.67	GC
103	MOL000239	Jaranol	314.31	50.83	0.29	GC
104	MOL002565	Medicarpin	270.30	49.22	0.34	GC
106	MOL003656	Lupiwighteone	338.38	51.64	0.37	GC
108	MOL000417	Calycosin	284.28	47.75	0.24	GC
111	MOL004806	euchrenone	406.56	30.29	0.57	GC
112	MOL004808	glyasperin B	370.43	65.22	0.44	GC
113	MOL004810	glyasperin F	354.38	75.84	0.54	GC
114	MOL004811	Glyasperin C	356.45	45.56	0.40	GC
115	MOL004814	Isotrifoliol	298.26	31.94	0.42	GC
116	MOL004820	kanzonols W	336.36	50.48	0.52	GC
117	MOL004827	Semilicoisoflavone B	352.36	48.78	0.55	GC
118	MOL004828	Glepidotin A	338.38	44.72	0.35	GC
119	MOL004829	Glepidotin B	340.40	64.46	0.34	GC
120	MOL004833	Phaseolinisoflavan	324.40	32.01	0.45	GC
121	MOL004835	Glypallichalcone	284.33	61.60	0.19	GC
122	MOL004841	Licochalcone B	286.30	76.76	0.19	GC
123	MOL004848	licochalcone G	354.43	49.25	0.32	GC
124	MOL004855	Licoricone	382.44	63.58	0.47	GC
125	MOL004856	Gancaonin A	352.41	51.08	0.40	GC
126	MOL004857	Gancaonin B	368.41	48.79	0.45	GC
127	MOL004879	Glycerin	382.44	52.61	0.47	GC
128	MOL004882	Licocoumarone	340.40	33.21	0.36	GC
129	MOL004883	Licoisoflavone	354.38	41.61	0.42	GC
130	MOL004884	Licoisoflavone B	352.36	38.93	0.55	GC
131	MOL004885	licoisoflavanone	354.38	52.47	0.54	GC
132	MOL004907	Glyzaglabrin	298.26	61.07	0.35	GC
133	MOL004908	Glabridin	324.40	53.25	0.47	GC
134	MOL005020	dehydroglyasperins C	340.40	53.82	0.37	GC
135	MOL005017	Phaseol	336.36	78.77	0.58	GC
110	MOL000422	kaempferol	286.25	41.88	0.24	GC, BS, CH
105	MOL000354	isorhamnetin	316.28	49.60	0.31	GC, CH
109	MOL004328	naringenin	272.27	59.29	0.21	GC, CP
107	MOL000392	formononetin	268.28	69.67	0.21	GC, DS
76	MOL000173	wogonin	284.28	30.68	0.23	HQ
77	MOL002917	5,2′,6′-Trihydroxy-7,8-dimethoxyflavone	330.31	45.05	0.33	HQ
78	MOL000228	(2R)-7-hydroxy-5-methoxy-2-phenylchroman-4-one	270.30	55.23	0.20	HQ
79	MOL002714	baicalein	270.25	33.52	0.21	HQ
80	MOL002909	5,7,2,5-tetrahydroxy-8,6-dimethoxyflavone	376.34	33.82	0.45	HQ
81	MOL002910	Carthamidin	288.27	41.15	0.24	HQ
82	MOL002913	Dihydrobaicalin_qt	272.27	40.04	0.21	HQ
83	MOL002914	Eriodyctiol (flavanone)	288.27	41.35	0.24	HQ
84	MOL002915	Salvigenin	328.34	49.07	0.33	HQ
85	MOL002925	5,7,2′,6′-Tetrahydroxyflavone	286.25	37.01	0.24	HQ
86	MOL002928	oroxylin a	284.28	41.37	0.23	HQ
87	MOL002932	Panicolin	314.31	76.26	0.29	HQ
88	MOL002933	5,7,4′-Trihydroxy-8-methoxyflavone	300.28	36.56	0.27	HQ
89	MOL002934	NEOBAICALEIN	374.37	104.34	0.44	HQ
90	MOL002937	DIHYDROOROXYLIN	286.30	66.06	0.23	HQ
91	MOL000525	Norwogonin	270.25	39.40	0.21	HQ
92	MOL000552	5,2′-Dihydroxy-6,7,8-trimethoxyflavone	344.34	31.71	0.35	HQ
93	MOL000073	ent-Epicatechin	290.29	48.96	0.24	HQ
94	MOL001458	coptisine	320.34	30.67	0.86	HQ
95	MOL002897	epiberberine	336.39	43.09	0.78	HQ
96	MOL008206	Moslosooflavone	298.31	44.09	0.25	HQ
97	MOL012245	5,7,4′-trihydroxy-6-methoxyflavanone	302.30	36.63	0.27	HQ
98	MOL012246	5,7,4′-trihydroxy-8-methoxyflavanone	302.30	74.24	0.26	HQ
99	MOL012266	rivularin	344.34	37.94	0.37	HQ
136	MOL000449	Stigmasterol	412.77	43.83	0.76	SDH, HQ, CH, CS, DG, DZ
137	MOL000296	hederagenin	414.79	36.91	0.75	ZL
138	MOL001004	pelargonidin	271.26	37.99	0.21	ZBM
140	MOL004440	Peimisine	427.69	57.40	0.81	ZBM
141	MOL004443	Zhebeiresinol	280.30	58.72	0.19	ZBM
139	MOL008583	beta-sitosterol	414.69	36.91	0.75	ZBM, ZL, SDH, BS, HQ, CS, CX, DG, DZ, DH

### The “herb-ingredient-target-disease” network analysis

The “herb-ingredient-target-disease” network of YYHXD was constructed by integrating the drug-disease overlapping targets and their corresponding active ingredients and herbs. As shown in Figure 1B, the “herb-ingredient-target-disease” network contained 224 nodes (represented 14 herbs, 141 compounds and 69 targets) and 490 edges (indicated interactions between nodes). By performing topology analysis, 5 compounds with highest degree score were screened out from the “herb-ingredient-target-disease” network and sequentially ordered as follows: quercetin, senkyunone, wallichilide, mandenol, myricanone, which may play a key role in the network (Table 2).

**Table 2:** The key components and their topology data.

**No.**	**Name**	**Betweenness**	**Closeness**	**Degree**
1	quercetin	5911.589	0.393043	23
2	senkyunone	1709.991	0.264327	12
3	wallichilide	1709.027	0.249173	12
4	Mandenol	4327.476	0.36748	11
5	Myricanone	2782.216	0.248625	11
6	luteolin	2475.88	0.393043	11
7	beta-sitosterol	1662.629	0.358162	10
8	augustic-acid	1294.088	0.284277	10
9	FA	2706.633	0.391681	10
10	3-N-butyl-4,5-dihydrophthalide	3141.471	0.36748	10

### PPI network analysis and functional enrichment analysis

Using BisoGenet plugin, the PPI analysis of 69 overlapping targets was performed. After screening twice with degree ≥2-fold median, the PPI network was finally constructed (Figure 1C). The PPI network contained 200 nodes and 2768 edges. The top 10 hub genes identified by CytoHubba using MCC algorithm were: TP53, ACTB, HSP90AA1, MYC, Akt1, CTNNB1, EP300, MDM2, JUN, RPS27A (Figure 1D (a–f)). It suggested that these hub genes may be the key targets for the treatment of hepatic fibrosis with YYHXD.

Using MCODE, 4 modules were chosen from PPI network. Cluster 1 was the module with the highest MCODE score and was therefore considered the most critical module in the PPI network. Cluster 1 is a gene cluster with all hub genes and other genes as the core, containing 34 genes and 494 edges. The module genes in cluster 1 were chosen for subsequent functional enrichment analysis (Figure 1D (a–f)).

To investigate the biological characteristics of modular genes in cluster 1, GO annotation and KEGG pathway analyses were carried out. GO analysis revealed the 34 modular genes were enriched in BP terms, 106 CC terms, and 132 MF terms, (*P* < 0.05 and FDR <0.05). The BP involved in cellular response to oxidative stress, cellular response to chemical stress, response to oxidative stress, cellular response to reactive oxygen species, response to reactive oxygen species, etc., Notably, oxidative stress related biological processes are widely enriched. The CC terms included transcription regulator complex, RNA polymerase II transcription regulator complex, cyclin-dependent protein kinase holoenzyme complex, spindle, serine/threonine protein kinase complex and so on. In terms of MF, the common targets were mostly enriched in DNA-binding transcription factor binding, RNA polymerase II-specific DNA-binding transcription factor binding, ubiquitin-like protein ligase binding, activating transcription factor binding, ubiquitin protein ligase binding and so on. The top 10 terms in the three categories of GO annotation are displayed in Figure 1E.

In addition, the 34 modular genes were enriched in various KEGG pathways, such as JAK-STAT signaling pathway, PI3K-Akt signaling pathway, Cell cycle, FoxO signaling pathway, Apoptosis. The top 10 most significantly enriched KEGG pathways were shown in Figure 1F (a, b) and Table 3. In this study, we focused on the PI3K-Akt signaling pathway, and subsequent molecular docking and animal experiments were carried out to demonstrate that the PI3K-Akt signaling pathway is an important mechanism for the anti-liver fibrosis effect of the YYHXD.

**Table 3:** The genes enriched in PI3K-Akt pathway.

**Pathway**	***p*-value**	***p*.adjust**	***q*-value**	**geneID**	**Count**
PI3K-Akt signaling pathway	8.28E-07	2.87E-06	7.71E-07	TP53/AKT1/MYC/HSP90AA1/MAPK3/EGFR/MDM2/BRCA1/MAPK1/CDK2	10

### Molecular docking validation

The 5 crucial active molecules in the “herb-ingredient-target-disease” network (quercetin, senkyunone, wallichilide, mandenol, myricanone) and the target proteins which were identified as hub genes and enriched in PI3K-Akt pathway (MYC, TP53, Akt1, HSP90AA1) were selected for molecular docking verification. The molecular docking results showed that the 5 crucial active molecules of YYHXD matched well with the PI3K-Akt pathway related proteins. The binding affinity expressed as LibDock score was shown in the heat map (Figure 1G (e)). Representative molecular docking results for the target proteins (quercetin, senkyunone, wallichilide, mandenol, myricanone) and their most relevant key ligand compounds are shown in Figure 1G (a–d). The molecular docking results suggested that the YYHXD may have a direct impact on the PI3K-Akt signaling pathway.

### YYHXD alleviated histopathological changes in liver fibrosis rats

**Figure 2:**
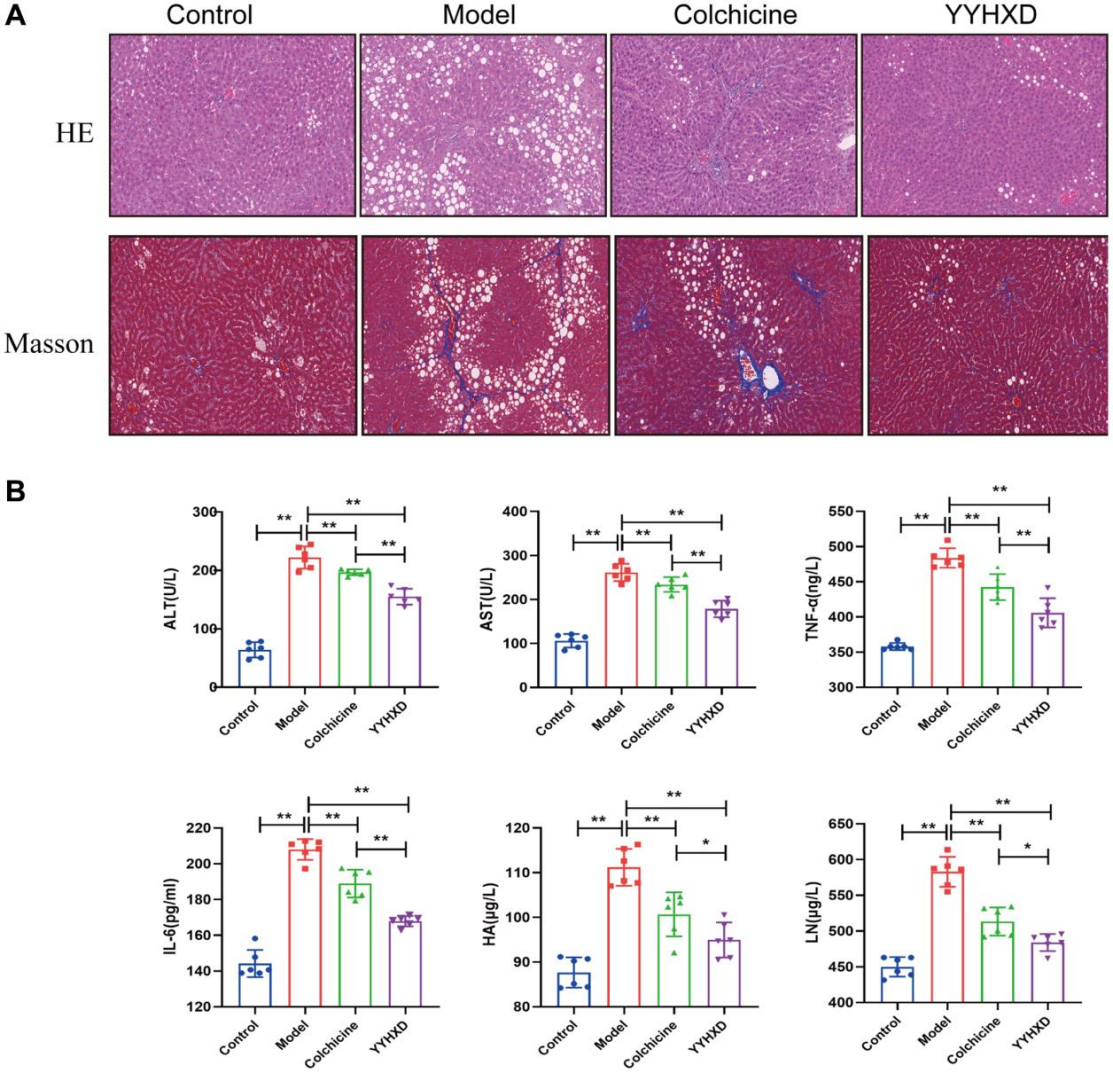
**Effect of YYHXD on histological changes in CCl4 rats.** (**A**) H&E staining (200X), Masson staining (200X) of rat livers. (**B**) Effect of YYHXD on level of inflammatory cytokines. Serum activities AST, ALT, IL6, TNF-α, HA and LN content. ^**^P < 0.01 compared with the model group.

The morphological changes of liver tissues were observed by HE and Masson staining, and the results were shown in Figure 2A. In the control group, the liver tissues showed normal lobular architecture and cellular structure. However, the liver tissues in the CCl_4_ model group exhibited lobular structure disarray, swelling and necrosis of hepatocytes, inflammatory cell infiltration, fatty degeneration, and pseudolobule formation. When compared to model group, these changes were less prominent in the YYHXD and colchicine treated groups. The results suggest that YYHXD can reduce the severity of liver fibrosis in CCl_4_-induced rats.

### YYHXD ameliorate serum indicators and hepatic α-SMA, and collagen I content in liver fibrosis rats

Serum ALT and AST were examined to indicate liver injury (Figure 2B). Compared to controls, CCl_4_ remarkably increased ALT and AST levels (*P* < 0.01), indicating severe liver damage. After YYHXD treatment, serum ALT and AST were significantly decreased compared with the model group (*P* < 0.01). After colchicine treatment, serum AST was significantly decreased compared with the model group (*P* < 0.01), which no significant change was observed in ALT level. Additionally, Serum ALT and AST levels were significantly lower in the YYHXD group than in the colchicine group (*P* < 0.01).

To assess inflammation, levels of IL-6 and TNF-α were examined (Figure 2B). CCl_4_ markedly upregulated IL-6 and TNF-α compared to controls. Both YYHXD and colchicine significantly inhibited the upregulation (*P* < 0.01). Furthermore, IL-6 and TNF-α levels were lower in YYHXD versus colchicine (*P* < 0.01).

To further evaluate the anti-fibrosis effect of YYHXD, serum HA and LN were measured (Figure 2B). The result showed that HA and LN levels were significantly increased in the CCl_4_ group compared with the normal control group (*P* < 0.01), and these markers were decreased remarkably in the YYHXD group and positive control group compared with the liver fibrosis model group (*P* < 0.01).

**Figure 3:**
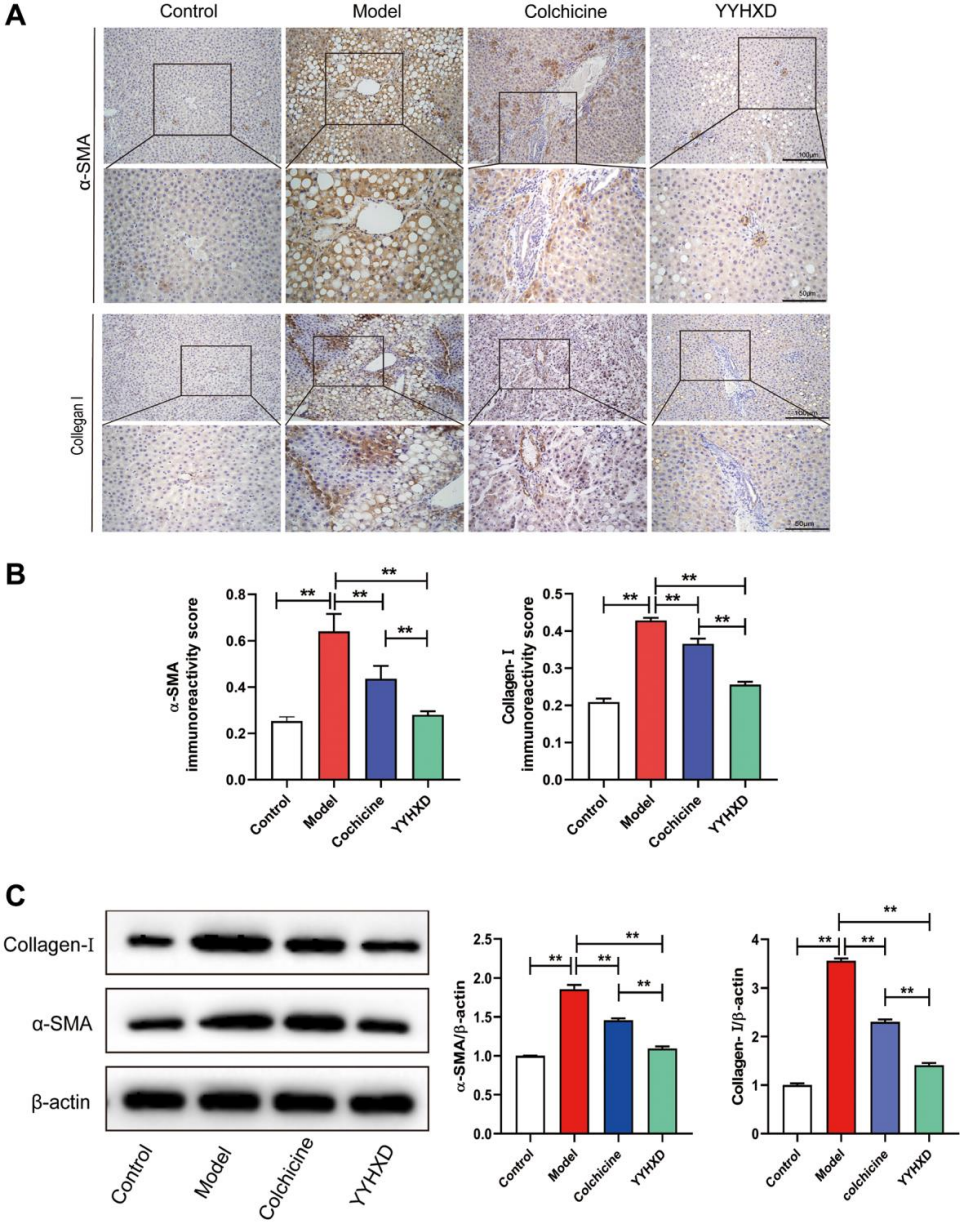
(**A**) a-SMA and collagen I expression in liver tissue were detected to investigate liver fibrosis. (**A**, **B**) Immunohistochemical images showed a-SMA and collagen I positive localization and positive expression rate. ^**^P < 0.01 compared with the model group. (**C**) Western blot image showing a-SMA and collagen I expression in liver tissues. ^**^P < 0.01 compared with the model group.

Furthermore, immunostaining revealed increased expression of α-SMA and collagen I, indicators of liver fibrosis, in CCl4 group compared to controls (*P* < 0.01). Both YYHXD and positive control significantly decreased α-SMA and collagen I expression versus CCl4 model (*P* < 0.01), suggesting YYHXD alleviated CCl_4_-induced fibrosis (Figure 3A–3C).

### YYHXD improved oxidative stress in liver fibrosis rats

**Figure 4:**
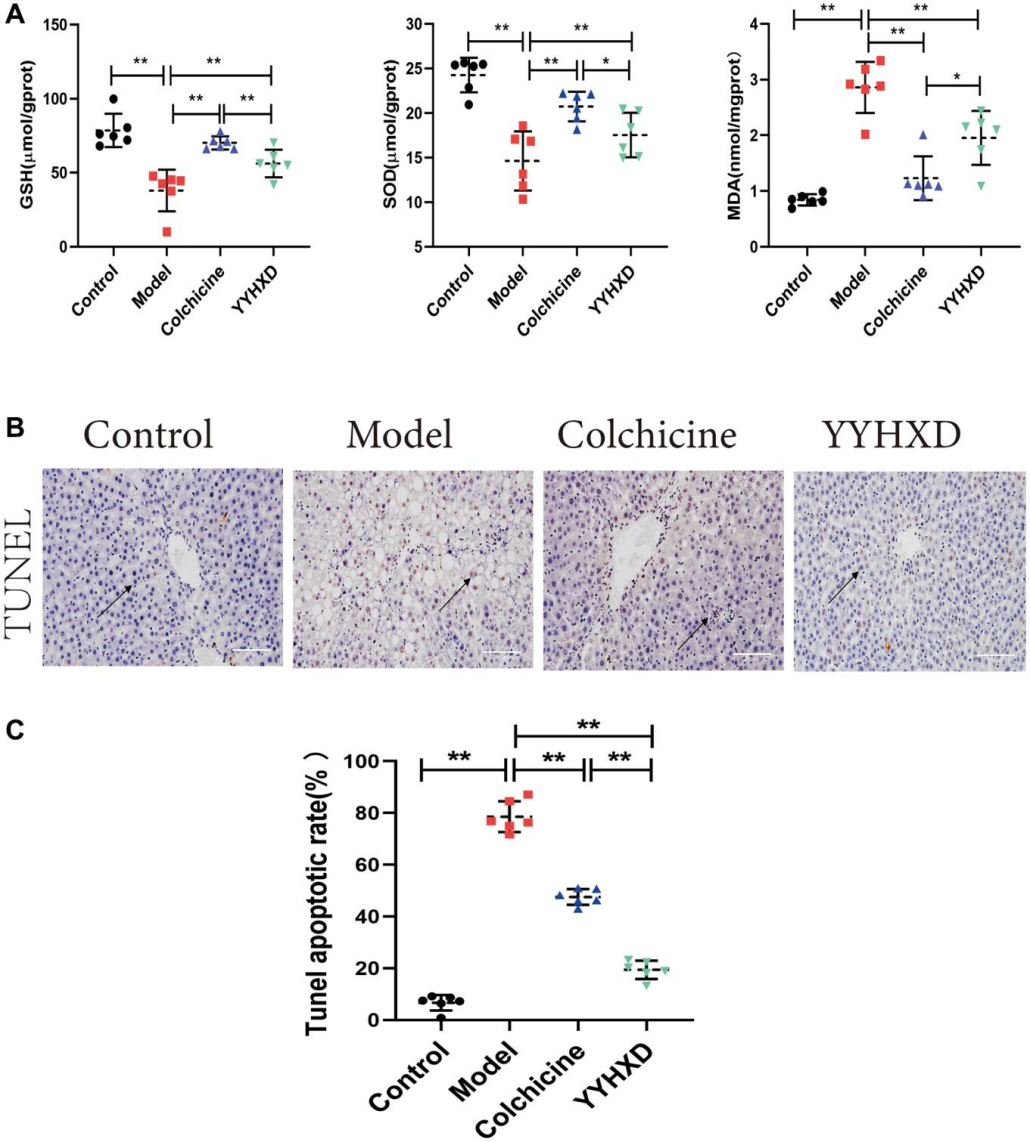
(**A**) Antioxidant levels in the treatment group YYHXD. GSH, SOD, MDA. (**B**, **C**) YYHXD can attenuate the apoptosis of liver parenchymal cells induced by CCl4 liver fibrosis, ^**^P < 0.01 compared with the model group. The result of TUNEL assay was shown in Figure 4B, 4C. The apoptosis rate in YYHXD group and colchicine group decreased significantly, ^****^P < 0.0001 compared with the model group.

GSH, SOD and MDA were measured to assess YYHXD antioxidant effects (Figure 4A). Compared with the normal group, the liver tissue GSH and SOD levels were significantly decreased and the levels of MDA were significantly increased in CCl_4_ model group (*P* < 0.01). Compared with the model group, the GSH and SOD levels were significantly increased and the levels of MDA were significantly decreased in the YYHXD group *(P* < 0.01). The colchicine group significantly decreased the MDA level (*P* < 0.01) but colchicine group has little impact on GSH and SOD compared with the model group. Overall, YYHXD can improve the antioxidant capacity, protecting the liver fibrosis induced by CCl_4_ in rats.

### YYHXD attenuated hepatocytes apoptosis in liver fibrosis rats

The result of TUNEL assay was shown in Figure 4B, 4C. The number of apoptotic liver parenchymal cells in the normal control group was very small, while more apoptotic hepatocytes were found in the liver tissue of the liver fibrosis model group. As expected, YYHXD could attenuated apoptosis of liver parenchymal cells induced by CCl_4_, Apoptosis rate in the blank group (0.07 ± 0.03) and the model group (0.79 ± 0.06), its ability to anti-apoptosis of hepatocytes was better than colchicine. It was obvious that the apoptosis rate in YYHXD group (0.19 ± 0.04) and colchicine group (0.48 ± 0.03) decreased significantly (*P* < 0.01), YYHXD group was significantly stronger than that in colchicine group (*P* < 0.01).

### YYHXD inhibited the PI3K-Akt signaling pathway in liver fibrosis rats

**Figure 5:**
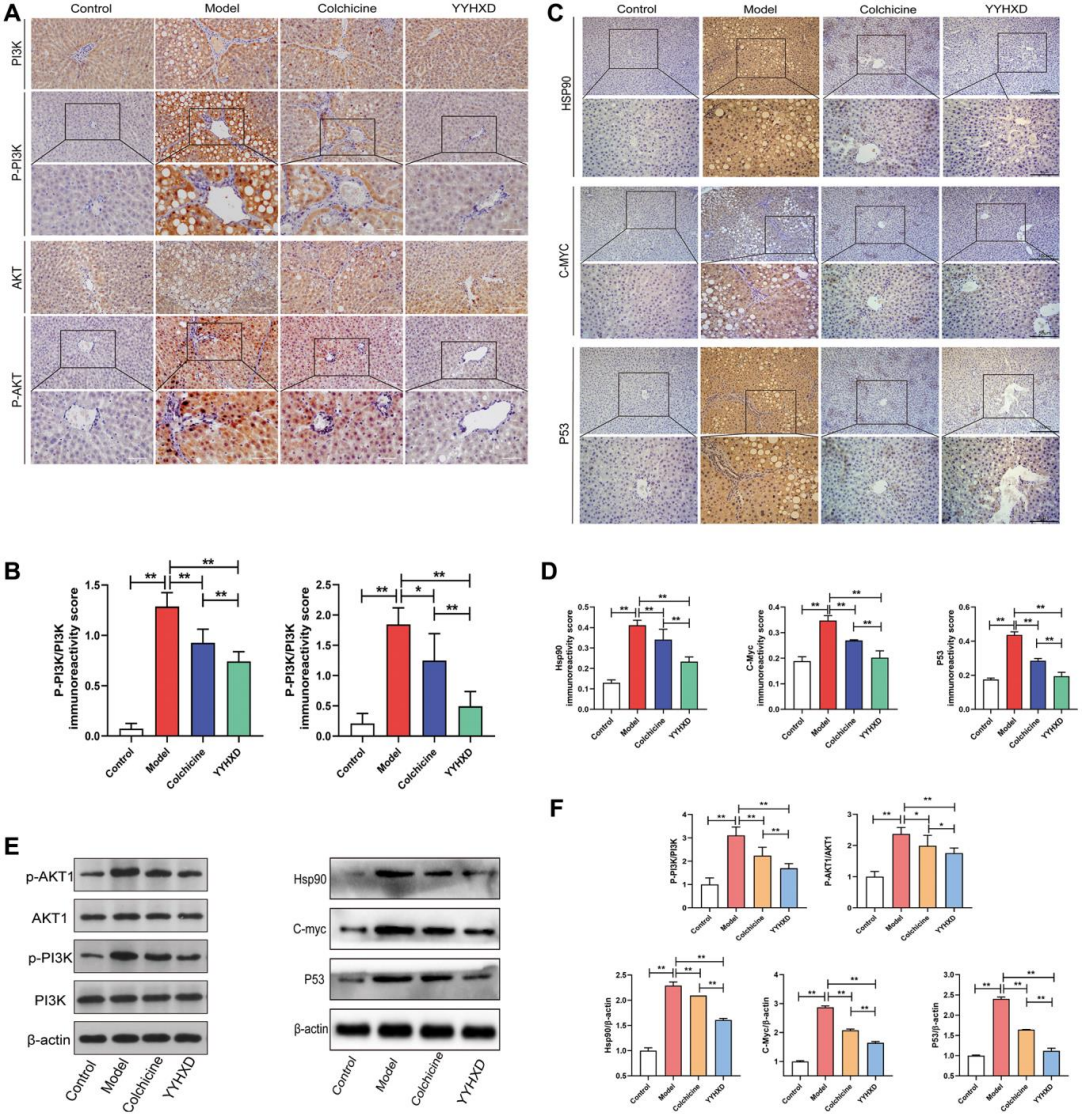
**PI3K-AKT signaling pathways and molecular docking targets.** (**A**, **C**) Immunohistochemical images showing P-PI3K, P-AKT1, HSP90, MYC, P53. Positive localization of protein in liver tissues. (**B**, **D**) Immunohistochemical positive expression rate. Expression rate. ^**^P < 0.01 compared with the model group. (**E**, **F**) Western blot image showing P-PI3K, P-AKT1, HSP90, MYC, P53 expression in liver tissues. ^**^P < 0.01 compared with the model group.

In this study, network pharmacology research and molecular docking experiments suggested that PI3K-Akt signaling pathway was potential pharmacological mechanism of YYHXD treatment for hepatic fibrosis. Thus, immunohistochemistry and Western blot assay were used to assess the effects of YYHXD treatment on the expression levels of the key genes in the pathway (P-PI3K, P-Akt1, HSP90, MYC, P53). As shown by Western blot assays and immunohistochemistry (Figure 5A–5D), the expression of P-PI3K, P-Akt1, HSP90, MYC, P53 proteins was upregulated in the CCl_4_ model group compared with the normal group (*P* < 0.01), while all were significantly attenuated by YYHXD and colchicine (*P* < 0.01). Moreover, compared with colchicine treated group, fewer expressions of P-PI3K, P-Akt1, HSP90, MYC, p53 proteins were found in YYHXD group by immunohistochemical detection (*P* < 0.01), and fewer expressions of P-PI3K, HSP90, p53 but not P-Akt1, MYC proteins were found in the YYHXD group compared to the colchicine treated group by Western blot analysis (*P* < 0.05) (Figure 5E, 5F).

## DISCUSSION

At present, traditional Chinese medicine is the most widely used complementary and alternative medical form [12, 13]. There is no doubt that traditional Chinese medicine plays an important role in disease treatment and human health. Because of its multi-component, multi-target and multi-channel characteristics, traditional Chinese medicine pays more attention to biological integrity, which is similar to the concept of network pharmacology [11]. Under the guidance of traditional Chinese medicine theory, YYHXD exerts the effect of soothing liver, regulating qi and promoting blood circulation. Clinically, YYHXD provides excellent safety and efficacy in the treatment of liver fibrosis. However, the pharmacological molecular mechanisms of YYHXD in the treatment of liver fibrosis is still unclear.

In this study, network pharmacology was utilized to explore YYHXD mechanisms against liver fibrosis. GO analysis revealed oxidative stress as a key process targeted by YYHXD. KEGG pathway analysis identified potential mechanisms including JAK-STAT, PI3K-Akt, cell cycle, FoxO and apoptosis pathways. Herb-component-target-disease network analysis identified five critical ingredients - quercetin, senkyunone, wallichilide, mandenol and myricanone - that may play pivotal roles in YYHXD’s anti-fibrotic effects.

Quercetin, being a natural antioxidant with preventive and therapeutic effects on liver fibrosis, is widely found in a variety of herbal medicines, including *bupleurum* [14], *tangerine peel* [15], *rhubarb* [16], *Scutellaria baicalensis* [17], *licorice* [18], etc. Wu et al. found that quercetin could prevent liver fibrosis by inhibiting TGF-β1/Smads and PI3K/Akt pathways to inhibit hepatic stellate cell activation and reduce autophagy [19]. A research of Aslam et al. indicated that quercetin ameliorated oxidative stress antagonizing the Hedgehog signaling pathway in treatment of hepatic fibrosis [20]. *Ligusticum wallichii* is an important Chinese herbal medicine in the composition of Yangyinghuoxue decoction, and has been proven to improve liver fibrosis by reducing oxidative stress [21]. Kang et al. revealed that Chuanxiong improved the inflammatory response in atherosclerotic mice by suppressing the PI3K-Akt signaling pathway [22]. Xu et al. predicted that Chuanxiong inhibited the activation of PI3K-Akt signaling pathway, and its active ingredients acted on multiple targets including INS, BDNF, FOS, VEGFA, PTGS2, ESR1, MAPK14 and so on based on network pharmacology and molecular docking research [23]. Therefore, senkyunone and wallichilide, being important components of pharmacodynamic material bases of Chuanxiong, may play the same anti-liver fibrosis pharmacological role as Chuanxiong. However, no studies have yet shown whether senkyunone and wallichilide target the PI3k-Akt signaling pathway as well as Chuanxiong. Here in this study, molecular docking experiments were used to initially validate the reliability of network pharmacology prediction results. The molecular docking results showed that quercetin, senkyunone, wallichilide, mandenol, and myricanone, the main active ingredients in YYHXD, targeted the PI3K-Akt signaling pathway and bound well to PI3K-Akt signaling pathway proteins, consistent with network pharmacology.

Liver fibrosis results from chronic injury and is characterized by ECM accumulation and inflammation [24]. This study demonstrated that YYHXD treatment could significantly decrease the elevated serum levels of aminotransferases (ALT/AST), proinflammatory cytokines (IL-6/TNF-α) and fibrosis biomarkers (HA/LN) in CCl4-induced fibrotic rat models. YYHXD also reduced excessive apoptosis in hepatic tissue, and ameliorated liver injury and collagen deposition. These results demonstrate significant anti-fibrotic effects of YYHXD, consistent with clinical observations.

Oxidative stress is a key driver of liver fibrosis. Endoplasmic reticulum and mitochondrial dysfunction can increase reactive oxygen species (ROS) production, aggravating mitochondrial damage [24]. Hepatic Kupffer cells are major sources of ROS upon toxic stimuli [25, 26]. ROS induce apoptosis and necrosis of hepatocytes, and thus regulates the hepatocyte cell cycle. Hepatocyte death releases mediators like TNF-α, IL-6 and TGF-β that stimulate inflammatory and fibrotic responses in neighboring hepatocytes, hepatic stellate cells and Kupffer cells [27, 28]. Additionally, ROS activate and proliferate hepatic stellate cells, stimulating ECM overproduction that disrupts normal liver structure and function. [26]. In this study, by detecting the GSH, SOD and MDA levels in liver tissues, we found that YYHXD significantly attenuated oxidative stress in liver tissue of hepatic fibrosis rats.

The phosphoinositide 3-kinase (PI3K)/protein kinase B (Akt) pathway (PI3K/Akt pathway) is one of the most significant intracellular signaling pathway which is documented to regulate biological process including oxidative stress, inflammatory reaction, cell growth, proliferation and survival, etc., [29]. The pathway is initiated by the binding of extracellular growth factors or cytokines to their corresponding cell surface receptors such as receptor tyrosine kinases (RTKs) or G protein-coupled receptors (GPCRs), leading to the activation of PI3K. This recruits and activates Akt, which phosphorylates downstream targets by translocating to cellular compartments including the nucleus, mitochondria and endoplasmic reticulum [30, 31]. Dysregulation of the PI3K/Akt pathway has been implicated in various diseases, including cancer, diabetes, cardiovascular disease, and neurodegenerative disorders [31]. Yu et al. have shown that the overactivation of the PI3K/Akt pathway is a hallmark of human malignancies, and PI3K inhibitors such as copanlisib, alpelisib, idelalisib, duvelisib, and umbralisib have been approved by the FDA for the treatment of various tumor diseases [32]. Mutations in the catalytic subunit p110α of PI3K have a high incidence in multiple cancers [32]. Dwivedi et al. have demonstrated that the activation of the PI3K/Akt pathway blocks cell apoptosis, promotes cell proliferation, increases the number of mature granulocytes, and exacerbates inflammatory reactions [33]. Activated PI3K signaling induces liver injury responses including oxidative stress, immune cell infiltration, hepatocyte death, stellate cell activation and excessive ECM deposition [34–36]. Therefore, suppression of PI3K-Akt signaling pathway is an effective way to treat liver fibrosis. In the current study, PI3K, Akt, HSP90, MYC, and p53 were identified as key targets for the treatment of hepatic fibrosis with YYHXD, and these targets were enriched in the PI3K/Akt signaling pathway. Through molecular docking experiments and animal experiments, the results conclusively demonstrated that YYHXD could target these target proteins and inhibit the activation of PI3K/Akt pathway.

## CONCLUSIONS

In this study, we applied network pharmacology approaches to systematically predict the mechanisms underlying YYHXD’s therapeutic effects on liver fibrosis. Pathway enrichment analysis indicated that the PI3K/Akt signaling pathway is a major target of YYHXD. Further experimental validation in a rat model revealed that YYHXD could inhibit PI3K/Akt activation, thereby exerting antioxidant and anti-apoptotic effects to alleviate liver fibrosis. Our integrative approach combining network pharmacology prediction and experimental verification highlights the clinical potential of YYHXD for treating liver fibrosis by targeting PI3K/Akt signaling.

## References

[1] Zhang CY, Yuan WG, He P, Lei JH, Wang CX. Liver fibrosis and hepatic stellate cells: Etiology, pathological hallmarks and therapeutic targets. World J Gastroenterol. 2016; 22:10512–22. 10.3748/wjg.v22.i48.10512 28082803 PMC5192262

[2] Dawood RM, El-Meguid MA, Salum GM, El Awady MK. Key Players of Hepatic Fibrosis. J Interferon Cytokine Res. 2020; 40:472–89. 10.1089/jir.2020.0059 32845785

[3] Parola M, Pinzani M. Liver fibrosis: Pathophysiology, pathogenetic targets and clinical issues. Mol Aspects Med. 2019; 65:37–55. 10.1016/j.mam.2018.09.002 30213667

[4] Moon AM, Singal AG, Tapper EB. Contemporary Epidemiology of Chronic Liver Disease and Cirrhosis. Clin Gastroenterol Hepatol. 2020; 18:2650–66. 10.1016/j.cgh.2019.07.060 31401364 PMC7007353

[5] Ginès P, Castera L, Lammert F, Graupera I, Serra-Burriel M, Allen AM, Wong VW, Hartmann P, Thiele M, Caballeria L, de Knegt RJ, Grgurevic I, Augustin S, et al, and LiverScreen Consortium Investigators. Population screening for liver fibrosis: Toward early diagnosis and intervention for chronic liver diseases. Hepatology. 2022; 75:219–28. 10.1002/hep.32163 34537988

[6] Cheng M, Feng X, Wang L, Yang Y, Ma L, Wang B. Nucleoside analogs assisted with Chinese compound prescription in treating hepatic fibrosis of chronic hepatitis B patients: A protocol of systematic review and meta-analysis. Medicine (Baltimore). 2020; 99:e21032. 10.1097/MD.0000000000021032 32629728 PMC7337550

[7] Liver Disease Committee, Chinese Association of Integrative Medicine. [Guidelines for the diagnosis and treatment of liver fibrosis with integrated traditional Chinese and Western medicine (2019 edition)]. Zhonghua Gan Zang Bing Za Zhi. 2019; 27:494–504. 10.3760/cma.j.issn.1007-3418.2019.07.005 31357774 PMC12769049

[8] Xu LM, Liu P, and Hepatology Committee of Chinese Association of Integrative Medicine, China. Guidelines for diagnosis and treatment of hepatic fibrosis with integrated traditional Chinese and Western medicine (2019 edition). J Integr Med. 2020; 18:203–13. 10.1016/j.joim.2020.03.001 32331978

[9] Zhou B, Wang G. Effect and Mechanism of YYHXT on Liver Fibrosis in Rats. Lishizhen Med Mater Med Res. 2018; 29:3.

[10] Yuan Z, Pan Y, Leng T, Chu Y, Zhang H, Ma J, Ma X. Progress and Prospects of Research Ideas and Methods in the Network Pharmacology of Traditional Chinese Medicine. J Pharm Pharm Sci. 2022; 25:218–26. 10.18433/jpps32911 35760072

[11] Jiashuo WU, Fangqing Z, Zhuangzhuang LI, Weiyi J, Yue S. Integration strategy of network pharmacology in Traditional Chinese Medicine: a narrative review. J Tradit Chin Med. 2022; 42:479–86. 10.19852/j.cnki.jtcm.20220408.003 35610020 PMC9924699

[12] Wang Y, Lou XT, Shi YH, Tong Q, Zheng GQ. Erxian decoction, a Chinese herbal formula, for menopausal syndrome: An updated systematic review. J Ethnopharmacol. 2019; 234:8–20. 10.1016/j.jep.2019.01.010 30658181

[13] Franco JVA, Turk T, Jung JH, Xiao YT, Iakhno S, Tirapegui FI, Garrote V, Vietto V. Pharmacological interventions for treating chronic prostatitis/chronic pelvic pain syndrome: a Cochrane systematic review. BJU Int. 2020; 125:490–6. 10.1111/bju.14988 31899937

[14] Shu-Chen G, Rui G, Yi-Teng X, Ting-Xia D, Huai-You W, Wah-Keung Karl T. [Quantitative analysis of fermented aerial part of Bupleurum chinense and prediction of their antimicrobial activity]. Zhongguo Zhong Yao Za Zhi. 2020; 45:4238–45. 10.19540/j.cnki.cjcmm.20200622.305 33164409

[15] Li X, Huang Y, Chen D. Protective Effect against Hydroxyl-induced DNA Damage and Antioxidant Activity of Citri reticulatae Pericarpium. Adv Pharm Bull. 2013; 3:175–81. 10.5681/apb.2013.029 24312832 PMC3846041

[16] Tan L, Geng DD, Hu FZ, Dong Q. Rapid Identification and Quantification of Natural Antioxidants in the Seeds of Rhubarb from Different Habitats in China Using Accelerated Solvent Extraction and HPLC-DAD-ESI-MSn-DPPH Assay. J Chromatogr Sci. 2016; 54:48–57. 10.1093/chromsci/bmv105 26206792

[17] Wang Z, Du Q, Qiu X, Liu F, Tan F, Lan K, Jiang X, Jiang Q. Simultaneous determination of six herbal components in intestinal perfusate by high-performance liquid chromatography. Biomed Chromatogr. 2009; 23:798–803. 10.1002/bmc.1188 19309764

[18] Sharma R, Gatchie L, Williams IS, Jain SK, Vishwakarma RA, Chaudhuri B, Bharate SB. Glycyrrhiza glabra extract and quercetin reverses cisplatin resistance in triple-negative MDA-MB-468 breast cancer cells via inhibition of cytochrome P450 1B1 enzyme. Bioorg Med Chem Lett. 2017; 27:5400–3. 10.1016/j.bmcl.2017.11.013 29150398

[19] Wu L, Zhang Q, Mo W, Feng J, Li S, Li J, Liu T, Xu S, Wang W, Lu X, Yu Q, Chen K, Xia Y, et al. Quercetin prevents hepatic fibrosis by inhibiting hepatic stellate cell activation and reducing autophagy via the TGF-β1/Smads and PI3K/Akt pathways. Sci Rep. 2017; 7:9289. 10.1038/s41598-017-09673-5 28839277 PMC5571156

[20] Aslam A, Sheikh N, Shahzad M, Saeed G, Fatima N, Akhtar T. Quercetin ameliorates thioacetamide-induced hepatic fibrosis and oxidative stress by antagonizing the Hedgehog signaling pathway. J Cell Biochem. 2022; 123:1356–65. 10.1002/jcb.30296 35696520

[21] Fu K, Wang C, Ma C, Zhou H, Li Y. The Potential Application of Chinese Medicine in Liver Diseases: A New Opportunity. Front Pharmacol. 2021; 12:771459. 10.3389/fphar.2021.771459 34803712 PMC8600187

[22] Kang Q, Liu W, Liu H, Zhou M. Effect of Compound Chuanxiong Capsule on Inflammatory Reaction and PI3K/Akt/NF-κB Signaling Pathway in Atherosclerosis. Evid Based Complement Alternat Med. 2015; 2015:584596. 10.1155/2015/584596 26539229 PMC4619937

[23] Xu L, Huang F, Zhang Y, Niu W, Pang J, Li S, Li X. [*Chuanxiong Rhizoma* inhibits brain metastasis of lung cancer through multiple active ingredients acting on multiple targets, pathways and biological functions]. Nan Fang Yi Ke Da Xue Xue Bao. 2021; 41:1319–28. 10.12122/j.issn.1673-4254.2021.09.05 34658345 PMC8526315

[24] Ramos-Tovar E, Muriel P. Molecular Mechanisms That Link Oxidative Stress, Inflammation, and Fibrosis in the Liver. Antioxidants (Basel). 2020; 9:1279. 10.3390/antiox9121279 33333846 PMC7765317

[25] Dixon LJ, Barnes M, Tang H, Pritchard MT, Nagy LE. Kupffer cells in the liver. Compr Physiol. 2013; 3:785–97. 10.1002/cphy.c120026 23720329 PMC4748178

[26] Luangmonkong T, Suriguga S, Mutsaers HAM, Groothuis GMM, Olinga P, Boersema M. Targeting Oxidative Stress for the Treatment of Liver Fibrosis. Rev Physiol Biochem Pharmacol. 2018; 175:71–102. 10.1007/112_2018_10 29728869

[27] Sánchez-Valle V, Chávez-Tapia NC, Uribe M, Méndez-Sánchez N. Role of oxidative stress and molecular changes in liver fibrosis: a review. Curr Med Chem. 2012; 19:4850–60. 10.2174/092986712803341520 22709007

[28] Parola M, Robino G. Oxidative stress-related molecules and liver fibrosis. J Hepatol. 2001; 35:297–306. 10.1016/s0168-8278(01)00142-8 11580156

[29] Jafari M, Ghadami E, Dadkhah T, Akhavan-Niaki H. PI3k/AKT signaling pathway: Erythropoiesis and beyond. J Cell Physiol. 2019; 234:2373–85. 10.1002/jcp.27262 30192008

[30] Huang X, Liu G, Guo J, Su Z. The PI3K/AKT pathway in obesity and type 2 diabetes. Int J Biol Sci. 2018; 14:1483–96. 10.7150/ijbs.27173 30263000 PMC6158718

[31] Fruman DA, Chiu H, Hopkins BD, Bagrodia S, Cantley LC, Abraham RT. The PI3K Pathway in Human Disease. Cell. 2017; 170:605–35. 10.1016/j.cell.2017.07.029 28802037 PMC5726441

[32] Yu M, Chen J, Xu Z, Yang B, He Q, Luo P, Yan H, Yang X. Development and safety of PI3K inhibitors in cancer. Arch Toxicol. 2023; 97:635–50. 10.1007/s00204-023-03440-4 36773078 PMC9968701

[33] Dwivedi P, Greis KD. Granulocyte colony-stimulating factor receptor signaling in severe congenital neutropenia, chronic neutrophilic leukemia, and related malignancies. Exp Hematol. 2017; 46:9–20. 10.1016/j.exphem.2016.10.008 27789332 PMC5241233

[34] Gong Z, Lin J, Zheng J, Wei L, Liu L, Peng Y, Liang W, Hu G. Dahuang Zhechong pill attenuates CCl4-induced rat liver fibrosis via the PI3K-Akt signaling pathway. J Cell Biochem. 2020; 121:1431–40. 10.1002/jcb.29378 31502329

[35] Zhao Y, Liu X, Ding C, Gu Y, Liu W. Dihydromyricetin Reverses Thioacetamide-Induced Liver Fibrosis Through Inhibiting NF-κB-Mediated Inflammation and TGF-β1-Regulated of PI3K/Akt Signaling Pathway. Front Pharmacol. 2021; 12:783886. 10.3389/fphar.2021.783886 34867416 PMC8634482

[36] Wang R, Song F, Li S, Wu B, Gu Y, Yuan Y. Salvianolic acid A attenuates CCl(4)-induced liver fibrosis by regulating the PI3K/AKT/mTOR, Bcl-2/Bax and caspase-3/cleaved caspase-3 signaling pathways. Drug Des Devel Ther. 2019; 13:1889–900. 10.2147/DDDT.S194787 31213776 PMC6549412

